# Zn-BTC MOF as Self-Template to Hierarchical ZnS/NiS_2_ Heterostructure with Improved Electrochemical Performance for Hybrid Supercapacitor

**DOI:** 10.3390/nano14010022

**Published:** 2023-12-20

**Authors:** Xuan Li, Lingran Liu, Chengyu Tu, Quan Zhang, Xinchun Yang, Daniil I. Kolokolov, Hanna Maltanava, Nikita Belko, Sergey Poznyak, Michael Samtsov, Haixin Guo, Shuping Wu, Maiyong Zhu

**Affiliations:** 1 Research School of Polymeric Materials, School of Materials Science & Engineering, Jiangsu University, Zhenjiang 212013, China; 2211905029@stmail.ujs.edu.cn (X.L.); 2222105022@stmail.ujs.edu.cn (L.L.); 2221905054@stmail.ujs.edu.cn (C.T.); shupingwu@ujs.edu.cn (S.W.); 2 State Key Laboratory for Modification of Chemical Fibers and Polymer Materials, College of Materials Science and Engineering, Donghua University, Shanghai 201620, China; 3 Clean Energy Joint International Laboratory, Low-Dimensional Energy Materials Research Center, Shenzhen Institute of Advanced Technology, Chinese Academy of Sciences, Shenzhen 518055, China; xc.yang@siat.ac.cn; 4 Boreskov Institute of Catalysis, Siberian Branch of Russian Academy of Sciences, Novosibirsk 630090, Russia; kdi@catalysis.ru; 5 Research Institute for Physical Chemical Problems, Belarusian State University, Leningradskaya Str. 14, 220006 Minsk, Belarus; maltanava@bsu.by (H.M.); belkonv@bsu.by (N.B.); poznyak@bsu.by (S.P.); samtsov@bsu.by (M.S.); 6 Agro-Environmental Protection Institute, Ministry of Agriculture and Rural Affairs, No. 31 Fukang Road, Nankai District, Tianjin 300191, China; haixin_g@126.com

**Keywords:** metal organic framework, metal sulfide, heterostructure, supercapacitor

## Abstract

Zn-BTC (H3BTC refers to 1, 3, 5-benzoic acid) MOF was used as a self-template and a zinc source to prepare ZnS/NiS_2_ with a layered heterogeneous structure as a promising electrode material using cation exchange and solid-phase vulcanization processes. The synergistic effect of the two metal sulfides enhances the application of ZnS/NiS_2_. And the high specific surface area and abundant active sites further promote the mass/charge transfer and redox reaction kinetics. In the three-electrode system, the specific capacitance was as high as 1547 F/g at a current density of 1 A/g, along with satisfactory rate capability (1214 F/g at 6 A/g) and cycling performance. Coupled with activated carbon (AC), the prepared hybrid device (ZnS/NiS_2_ as the positive electrode and AC as the negative electrode) (ZnS/NiS_2_/AC) can be operated under a potential window of 1.6 V and provides a high energy density of 26.3 Wh/kg at a power density of 794 W/kg. Notably, the assembled ZnS/NiS_2_//AC showed little capacity degradation after 5000 charge/discharge cycles.

## 1. Introduction

Hybrid supercapacitors, consisting of a capacitive electrode and battery electrode, are becoming a prominent alternative to chargeable battery and traditional carbon-based supercapacitors since they possess both high energy density and output power density. These inherent features make hybrid supercapacitors promising in the utilization of renewable energy. One major obstacle impeding the large-scale application of hybrid supercapacitors is the mismatch between a battery positive electrode and capacitive negative electrode in kinetics [1,2,3]. The current metal oxide battery electrodes face the bottleneck of low electronic conductivity and theoretical specific capacitance value, limiting the exploration of their further practical application [4,5,6,7]. Hence, there has been numerous research on non-oxide electrodes with excellent electronic conductivity and high capacitance, such as sulfides, nitrides and layered double hydroxides, etc., owing to their adjustable transition metal active sites and excellent mechanical and thermal stability [8,9,10,11].

Transition metal sulfides have become promising anode materials for supercapacitors by virtue of their good electronic conductivity, strong redox reversibility and low cost. Among them, ZnS, as a wide bandgap semiconductor material with the advantages of high electrical conductivity, strong stability and easy preparation, has been widely used in hybrid supercapacitor anode materials [12]. For example, as early as in 2006, Jayalakshmi et al. reported that ZnS nanoparticles synthesized via a solvothermal process using thiourea as a sulfur source were ideal to be utilized as electrodes in supercapacitors [13]. Through the hydrothermal technique and anion exchange reaction, Yi et al. synthesized ZnS nanoparticles manifesting a supreme reversible specific capacitance, outstanding rate capability and prominent ultralong cycle life [14]. Besides pristine ZnS, some composites containing ZnS, such as ZnS/graphene and ZnS/g-C_3_N_4_, were also reported to deliver reversible capacitance [15,16]. In spite of the great achievements of the ZnS-based electrodes with improved electrochemical performance, the capacitances of many previous reports are still far away from the practical requirements. Moreover, the same as other monometal sulfides, the limited number of electrochemically active sites, large volume expansion and low conductivity form vital challenges for exploring the full potential of ZnS as a supercapacitor electrode. Bimetal sulfides are expected to show better electrochemical properties than monometal sulfides.

Numerous efforts have been devoted to improve the electrochemical performance of the hybrid supercapacitor including optimizing the structure, composition and morphology of electrode material [17]. Among them, the construction of heterogeneous interfaces is considered to be an effective strategy to improve the electrochemical performance of electrode materials. It can enhance the structural stability of the material while promoting charge transfer within the electrode material, thus further accelerating the reaction kinetics. The construction of heterogeneous structures can prevent the structural crushing of ZnS and provide additional conduction pathways. In addition, the presence of two or more components in a heterostructure yields multiple electrochemically active sites and synergistic interactions, which may boost the overall electrochemical performance of the electrode, including rate capability and cycling stability. Javed et al. have synthesized porous 2D ZnS/FeS composites, which offer a capacitance (1367.5 F/g at 3 A/g) with an outstanding operation life [18]. Saeed et al. developed hybrid electrode materials in which core-shell Zn-Ni_7_S_6_ nanosheet arrays were wrapped with Ni(OH)_2_ nanopetals (ZnS-Ni_7_S_6_/Ni(OH)_2_), quoting a commendable area capacitance outcome of 13.55 F/cm^2^ at 5 mA/cm^2^ and a long cycling life of 95.12% maintenance rate of capacitance over 10,000 cycles [19]. Arul et al. have reported that the ZnS/MnS heterostructures delivered a specific capacitance of 884 F/g at 2 mV/s, which was much higher than that of pristine MnS (423 F/g at 2 mV/s) [20]. ZnS/Ni_3_S_2_ electrode material obtained by the chemical co-precipitation of Ni^2+^ and Zn^2+^ followed by sulfurization was demonstrated to express high area-specific capacitance (890.1 C/g at 1 A/g), good rate capability (70% retention of its initial value from 1 A/g to 20 A/g) and good cycling stability (82% retention of initial value after 6000 cycles at a current density of 10 A/g) [21]. From the above examples, it can be concluded that constructing a heterostructure with other metal sulfides can enable ZnS to achieve better electrochemical performance. The reason for this, to a great extent, is because it relies on the coexistence of different metal cations, which provides multiple valence transitions, enabling multiple redox reactions and enriching structural deficiencies.

Based on the above discussion, in this paper, hierarchical ZnS/NiS_2_ heterostructures were formed via cation exchange and solid-phase sulfidation treatments using Zn-BTC (H3BTC refers to 1,3,5-benzenetricarboxylic acid) MOF as a self-template [22,23]. The micron-scale rod-like structure was transformed into a nanoscale porous flower-like structure during the cation exchange process, and solid-phase sulfidation was an effective way to construct multilayered composite metal sulfides by obtaining both metal sulfide heterojunctions and introducing a porous carbon skeleton. The formation of heterostructures has an obvious role in promoting charge/ion transfer as well as conductivity enhancement. The presence of mesopores and macropores gives the synthesized ZnS/NiS_2_ a high specific surface area of 74.88 m^2^/g, which is conducive to the provision of active sites and the reduction in mass/charge transfer resistance. When tested as supercapacitor electrodes, the ZnS/NiS_2_ heterostructures produced a high mass specific capacitance of 1547 F/g at 1 A/g with excellent retention and attractive cycling stability. To demonstrate their practical application, the researchers further constructed a hybrid supercapacitor device (ZnS/NiS_2_//AC) by using the generated ZnS/NiS_2_ as the positive electrode and commercial activated carbon (AC) deposited on nickel foam as the negative electrode. The assembled device can operate within a voltage window of 1.6 V and produces an excellent energy density of 26.3 Wh/kg at a power density of 794 W/kg, with satisfactory cycling stability and capacitance retention close to 100% after 5000 cycles. This study opens new horizons for investigating the potential of MOF-derived heterostructures in improving electrochemical performance and optimizing the microstructure of materials.

## 2. Experimental Section

### 2.1. Chemicals and Reagents

Zinc acetate dehydrate (Zn(Ac)_2_·2H_2_O), nickel acetate tetrahydrate (Ni(Ac)_2_·4H_2_O), 1,3,5-Benzenetricarboxylic acid (H_3_BTC), nickel nitrate hexahydrate (Ni(NO_3_)_2_·6H_2_O), sulfur powder, potassium hydroxide (KOH), and absolute ethanol were supplied by Sinopharm Chemical Reagent Co., Ltd. All chemicals were used as received without further purification.

### 2.2. Material Preparation

#### 2.2.1. Synthesis of Zn-BTC Precursor Nanorods

A conventional solvothermal process was utilized to synthesize the Zn-BTC precursor nanorods. In particular, 2.1 mmol of Zn(Ac)_2_·2H_2_O was dissolved in 4.22 mL of water to form a clear solution. Then, 8.34 mL of ethanol dissolved in 2.5 mmol of H_3_BTC was added, yielding a homogeneous solution. In order to form Zn-BTC, the homogeneous solution was transferred into a 50 mL Teflon-lined stainless autoclave, which was placed in an oven and kept at 175 °C for 24 h. The prepared Zn-BTC was collected via centrifugation and washed with absolute ethanol and deionized water several times. In the end, the Zn-BTC was dried at 60 °C overnight. For further comparison, Zn(Ac)_2_·2H_2_O was replaced by Ni(Ac)_2_·4H_2_O, and the as-prepared green samples were named Ni-BTC.

#### 2.2.2. Synthesis of Zn/Ni-BTC

The Zn/Ni-BTC was synthesized via a simple cation exchange reaction. A total of 4 mmol of Ni(NO_3_)_2_·6H_2_O was dispersed into 30 mL of absolute ethanol via magnetic stirring. Then, 0.2 g of the obtained Zn-BTC powders was added to the suspension and stirred for 30 min. Then, the mixed solution was transferred into a 50 mL Teflon-lined stainless autoclave. The reaction was performed at 180 °C for 6 h. After cooling to room temperature, the resultant Zn/Ni-BTC powder was collected via centrifugation and washed several times with ethanol and deionized water before drying under a vacuum at 60 °C overnight.

#### 2.2.3. Synthesis of ZnS/NiS_2_

The ZnS/NiS_2_ was fabricated via a solid-phase vulcanization reaction. The as-prepared Zn/Ni-BTC was mixed via grounding with sulfur powder in a mass ratio (1:2). Then, the mixture was loaded into a quartz boat for vulcanization. The vulcanization was carried out in a tube furnace at 500 °C for 2 h with a heating rate of 5 °C/min in a N_2_ atmosphere. The final product was named ZnS/NiS_2._ For further comparison, the Zn-BTC and Ni-BTC were also treated using the above method, and the as-prepared samples were named ZnS and NiS.

### 2.3. Material Characterizations

The phase and crystal structure of the samples were determined using X-ray diffraction (XRD, D8 ADVANCE, BRUKER, Berlin, Germany) with a Cu target in the 10° to 90° range with a step size of 5°/min. The morphology of the surface and cross-section of the sintered electrolyte pellets were analyzed using a scanning electron microscope. The morphologies of the materials were characterized using field-emission scanning electron microscopy (FESEM, QUANTA FEG 250, Thermo, Amsterdam, Netherland) and transmission electron microscopy (TEM, JEM-2100, JEOL, Tokyo, Japan). An X-ray photoelectron spectrometer (XPS, AXIS Ultra DLD, Kratos, Tokyo, Japan) was utilized to determine the surface chemical compositions of ZnS/NiS_2_. The measurement of surface area was performed using a Quantachrome BET instrument. The specific surface area and porosity were determined using the Brunauer–Emmett–Teller (BET) equation based on N_2_ adsorption–desorption isotherms and the Barrett−Joyner−Halenda (BJH) model.

### 2.4. Electrochemical Testing

A conventional three-electrode system was initially utilized for electrochemical measurement, in which the counter electrode and reference electrode were platinum foil and KCl-saturated Hg/ Hg_2_Cl_2_, respectively, whereas ZnS/NiS_2_ samples on nickel foam were the working electrode. Specially, the working electrode was prepared as below. The slurry was prepared by taking 0.03 g of the prepared sample and then mixing the sample, carbon black and polyvinylidene fluoride (PVDF) in N methyl pyrrolidone (NMP) solvent in a mass ratio of 75:15:10. Then, the obtained slurry was uniformly spread on nickel foam. The fabricated electrode was dried in a vacuum oven at 60 °C overnight and pressed with a pressure of 10 MPa. And the loading mass of active materials was about 3–5 mg/cm^2^. 

The electrochemical testing was conducted on an electrochemical working station (CHI760E). The cyclic voltammetry (CV), galvanostatic charge–discharge (GCD) and electrochemical impedance spectroscopy (EIS) measurements were used to investigate the electrochemical properties and the electrolyte of 1 M KOH aqueous solution in the three-electrode system, in which the counter electrode and reference electrode were platinum foil and KCl-saturated Hg/ Hg_2_Cl_2_, respectively. In addition, the KOH/PVA gel was coated on the active substance area of the ZnS/NiS_2_ and AC electrodes, respectively, and the electrode assembly was carried out in the semi-dry state, and the hybrid supercapacitor was prepared by wrapping the tape after full solidification, so that the possibility of the practical application of the ZnS/NiS_2_ heterostructures could be examined. Similar procedures to a three-electrode system were utilized to prepare the corresponding positive and negative electrodes of the device. To balance the charges in both electrodes, the mass ratio of active materials loading on the positive (ZnS/NiS_2_) and negative electrode (active carbon) was about 1:5. The solid electrolyte of KOH/PVA was utilized in the hybrid device, which was prepared according to the authors’ previous report. The gravimetric specific capacitance (*Cs*) of active material with active mass (*m*) loading [*g*] was calculated using Equation (1)

(1) $$C_{S}=\frac{i\times \Delta t}{\Delta V\times m}\;\;(F/g)$$

The energy density (*E*) and power density (*P*) of the hybrid device were calculated using Equations (2) and (3):

(2) $$E=\frac{1}{2\times 3.6}\times \Delta V^{2}\times C\;\;\;(Wh/kg)$$

(3) $$P=\frac{E\times 3600}{\Delta t}\;\;(W/kg)$$

Herein, i refers to the discharge current density (A/g), Δ*t* is discharging time (s), m refers to the mass of active materials loaded on the electrode (g), Cs is the specific capacitance (*F*/*g*) and Δ*V* refers to the applied voltage window (*V*).

## 3. Results 

### 3.1. Synthesis Principle and Characterization of Materials

**Scheme 1:**
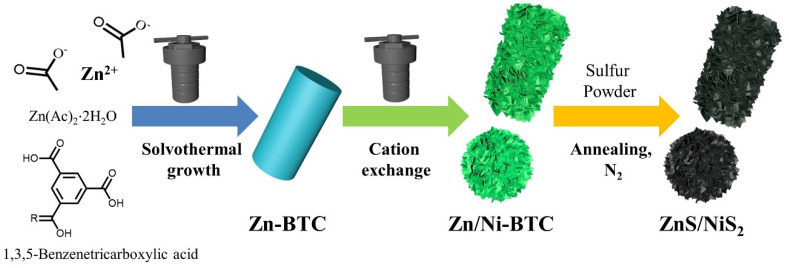
Schematic diagram of synthesis process of ZnS/NiS2.

The whole synthetic process of the ZnS/NiS_2_ heterostructure is illustrated in Scheme 1. Two solvothermal reactions and a one-step annealing procedure are involved. Firstly, Zn(Ac)_2_·2H_2_O and H_3_BTC were used as reactants in the first solvothermal procedure. Zn^2+^ reacted with H_3_BTC at a high temperature to form solid Zn-BTC [24]. Subsequently, bimetal Zn/Ni-BTC MOF could be obtained via cation exchange under the second solvothermal procedure. During this step, Ni^2+^ meets the solid Zn-BTC whereby the metal ion exchange occurs. According to good thermodynamics at high temperature, the reaction can be carried out effectively. Through the addition of Ni(NO_3_)_2_·6H_2_O to the Zn-BTC solution, the protons can be released by the hydrolysis of Ni^2+^ [25]. With the increase in hydrothermal time, more and more protons are produced to etch the Zn-BTC further. As a result, Zn^2+^ was partially replaced by Ni^2+^. At the same time, the BTC released into the solution may have recombined with unreacted Ni^2+^ as well as Zn^2+^ in the solution to produce Zn/Ni-BTC, which is a thermodynamic controlled process. The final consequence was that two types of Zn/Ni-BTC were possibly obtained in the system. Owing to the Kirkendall effect, the Zn/Ni-BTC possesses hierarchical porous structures. Finally, an annealing treatment was carried out. Owing to the presence of sulfur powder, the Zn/Ni-BTC MOF was easily converted to the ZnS/NiS_2_ heterostructure.

**Figure 1:**
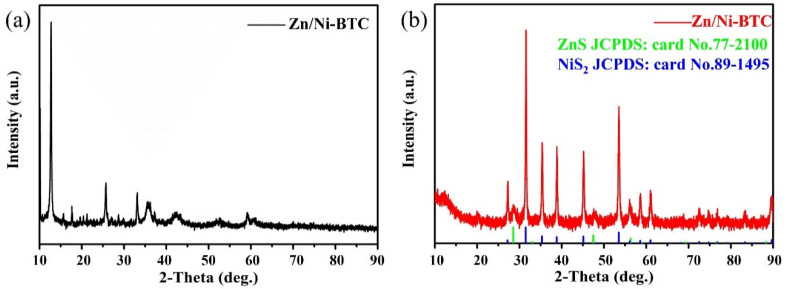
Typical wide-angle XRD patterns of Zn/Ni-BTC (**a**) and ZnS/NiS_2_ (**b**).

In order to better understand the synthesized ZnS/NiS_2_ heterostructure as well as its precursor, some characterization equipment was utilized for their compositions, morphologies as well as structures. The crystalline structure of Zn-BTC was confirmed via XRD (Figure S1a), in which all peaks were in good agreement with the literature report [24]. A further hydrothermal treatment on Zn-BTC in the presence of Ni(NO_3_)_2_ yielded Zn/Ni-BTC, the XRD pattern of which is shown in Figure 1a. The ZnS/NiS_2_ was obtained via the annealing of the Zn/Ni-BTC and sulfur powder. To elucidate the chemical composition, XRD was carried out to identify the crystal structure ZnS/NiS_2_. In agreement with the standard patterns (JCPDF #77-2100), signals of (111), (220) and (300) crystal planes of ZnS were observed at 28.5°, 47.5° and 56.3°, as shown in Figure 1b. The other peaks in ZnS/NiS_2_ were at 27.1°, 31.5°, 35.2°, 38.9°, 45.1°, 53.5°, 56.1°, 58.6° and 61.0°, which are indexed to (111), (200), (210), (211), (220), (331), (222), (023) and (321) crystal planes of NiS_2_ (JCPDF #89-1495). For comparison, the controlled sample of pristine ZnS was prepared via the direct sulfurization of Zn-BTC. The corresponding XRD of pristine ZnS is displayed in Figure S1b, and it confirms that the sample was ZnS. Additionally, Ni-BTC MOF was also prepared using a similar strategy with Zn-BTC and was further sulfided. As presented in Figure S2, the Ni-BTC exhibited good crystallinity. However, the sulfided product of Ni-BTC was confirmed to be NiS but not NiS_2_.

**Figure 2:**
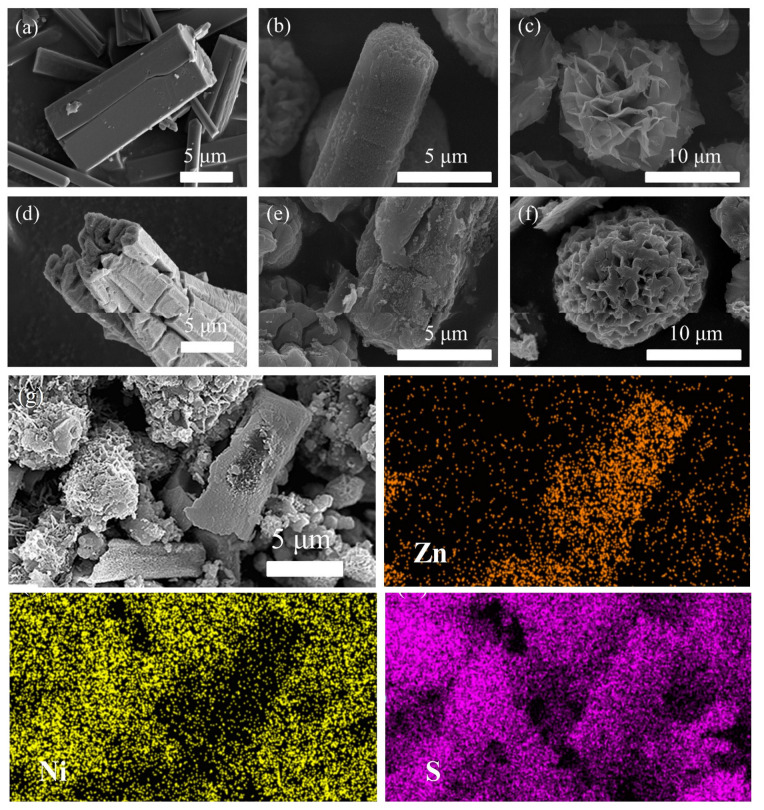
FESEM images of (**a**) Zn-BTC, (**b**,**c**) Zn/Ni-BTC, (**d**) ZnS, (**e**,**f**) ZnS/NiS_2_, and (**g**) typical SEM-EDX results for ZnS/NiS_2_.

**Figure 3:**
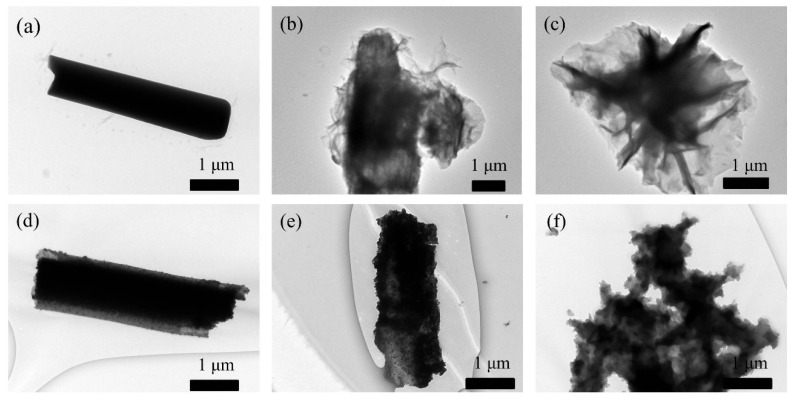
TEM images of (**a**) Zn-BTC, (**b**,**c**) Zn/Ni-BTC, (**d**) ZnS, (**e**,**f**) ZnS/NiS_2_.

Figure 2a presents a typical SEM image of the Zn-BTC, exhibiting the shape of a nanorod. Such a rod-like solid precursor is suitable as the sacrificial template for an active material’s growth and chemical transformation. Zn-BTC nanorods are approximately 2–10 µm in diameter, have a smooth surface and are highly oriented. Under a further solvothermal condition, Zn/Ni-BTC was synthesized vi a cation-exchange reaction between Ni^2+^ in the solution and Zn^2+^ in the as-obtained Zn-BTC. As presented in Figure 2b,c, Zn/Ni-BTC maintains the size and overall structure of the nanorods and gradually grows nanoflowers, which increases the specific surface area of the material and introduces a large distribution of mesopores. Field-emission scanning electron microscope (FESEM) images of ZnS and ZnS/NiS_2_ are presented in Figure 2d–f. After high-temperature annealing and vulcanization, the further decomposition of the organic ligands on the basis of the nanorods with nanoflowers results in a richer pore size distribution, which is more favorable for ion diffusion and transport. From the EDX mapping images (Figure 2g), the uniform distributions of Zn, Ni and S in the sample are the direct evidence of the successful synthesis of a ZnS/NiS_2_ heterostructure. The microstructures of ZnS and ZnS/NiS_2_ were further detected using a transmission electron microscope (TEM). As shown in Figure 3a,d, the dense nanorod structure starts to become rough and loose, with many mesopores and micropores, greatly increasing the number of electrochemical reaction sites. This structural transformation trend is also seen in the preparation of ZnS/NiS_2_ from Zn/Ni-BTC, which is shown in Figure 3b,c,e,f. In addition, the chemical composition and morphology of Ni-BTC and NiS are further studied and discussed. The morphology of Ni-BTC also presents as a nanorod (Figure S3), and the size is slightly smaller than that of Zn-BTC, which are detected as having a porous character.

**Figure 4:**
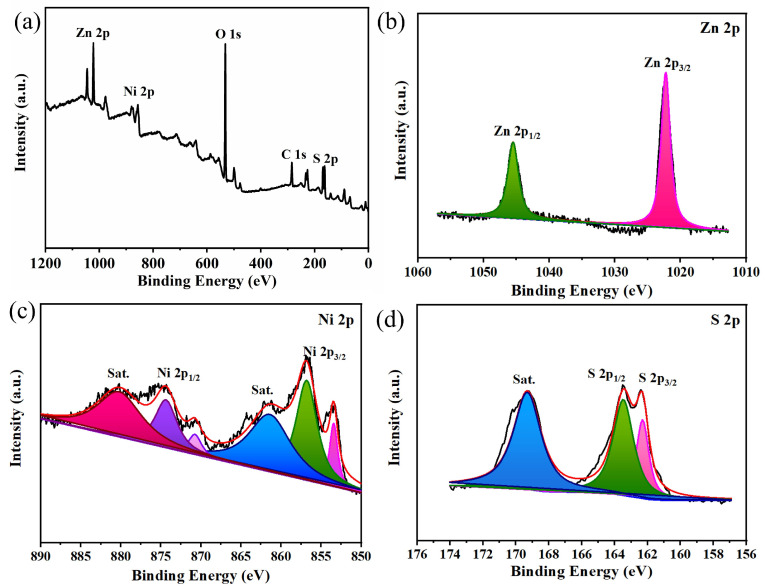
XPS spectra ZnS/NiS_2_ sample: (**a**) survey, (**b**) Zn 2p, (**c**) Ni 2p and (**d**) S 2p.

X-ray photoelectron spectroscopy (XPS) was applied to explore the surface electron states of ZnS/NiS_2_. As shown in Figure 4a, the characteristic peaks in the full measurement scanning spectrum confirmed the existence of Zn, Ni and S elements, which are consistent with XRD and EDX results. As revealed by Figure 4b, the Zn 2p exhibited two peaks of Zn 2p3/2 (1021.3 eV) and Zn 2p1/2 (1045.3 eV), which originated from ZnS [26]. Figure 4c presents the spectrum of Ni 2p with six peaks. The peaks centering at 853.1 eV and 871.4 eV can be indexed to Ni2p3/2 and Ni2p1/2 of Ni(II), while peaks at 857.2 eV 874.6 eV belong to Ni2p3/2 and Ni2p1/2 of Ni(III). Moreover, two satellite peaks (862.5 eV and 880.1 eV) were observed for Ni(II) and Ni(III), respectively [27,28]. A Ni(III)/Ni(II) ratio of 9:5 was found based on an analysis of XPS data. As far as S 1s is concerned, three peaks are observed, as shown in Figure 4d. There are two peaks corresponding to the S 2p_3/2_ and 2p_1/2_ orbitals of S at binding energies of 162.5 and 163.4 eV, respectively. This result evidences the existence of a metal–S (Zn-S and Ni-S) bond. Owing to the surface oxidation, the characteristic signal of S species with a high oxidation state is observed at 169.4 eV [29,30].

**Figure 5:**
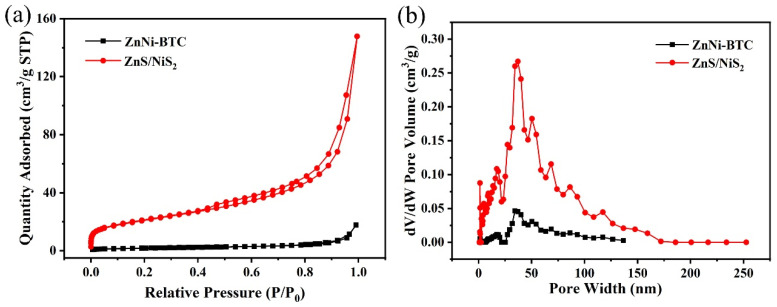
(**a**) Nitrogen adsorption/desorption isotherms for the Zn/Ni-BTC and ZnS/NiS_2_ electrodes. (**b**) The corresponding pore-size distribution curves obtained using the BJH method.

Both Zn/Ni-BTC and the final synthesized ZnS/NiS_2_ heterostructures are expected to be rich in porous structures. Thus nitrogen adsorption–desorption measurement was carried out to determine the specific surface areas and porosity of Zn/Ni-BTC and ZnS/NiS_2_. As shown in Figure 5a, ZnS/NiS_2_ possesses a much higher adsorption amount of N_2_ compared to Zn/Ni-BTC, revealing that ZnS/NiS_2_ exhibits a larger surface area than Zn/Ni-BTC. This result demonstrates that solid vulcanization under high temperature brings out more pores in the sample. Additionally, both curves could be ascribed to type IV. On the basis of the N_2_ adsorption–desorption isotherm curve, the BET surface areas of the ZnS/NiS_2_ and Zn/Ni-BTC are calculated to be 74.88 m^2^/g and 6.89 m^2^/g, respectively. The increase in the BET surface area could correspond to the decomposition of Zn/Ni-BTC materials at a high temperature and the formation of pore-rich sulfide, which were observed in the SEM and TEM images. As can be seen from Figure 5b, the pore size of Zn/Ni-BTC is mainly located in the range of micropores and mesopores, while the ZnS/NiS_2_ heterostructure has several larger pores. Such porous features are beneficial for mass/ion transfer with a high rate, which is critical for improving electrochemical reaction kinetics [31]. The high surface area of ZnS/NiS_2_ will provide rich defects and pores, which are conducive to accelerating electron transfer and the transport of reactants to active sites [32]. Thus, the specific capacitance and charge discharge rate capability are expected to be greatly improved.

### 3.2. Electrochemical Performance of Materials

**Figure 6:**
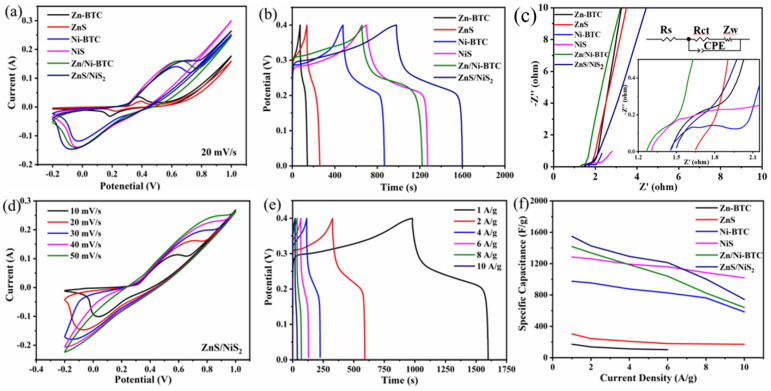
Electrochemical behaviors of Zn-BTC, ZnS, Ni-BTC, NiS_,_ Zn/Ni-BTC and ZnS/NiS_2_ electrodes. (**a**) CV profiles at 20 mV/s. (**b**) GCD profiles at 1 A/g. (**c**) EIS plots. (**d**) CV profiles of ZnS/NiS_2_ within 10–50 mV/s. (**e**) GCD curves of ZnS/NiS_2_ within 1–10 A/g current density. (**f**) Plots of specific capacitance vs. current density of Zn-BTC, ZnS, Ni-BTC, NiS_,_ ZnNi-BTC and ZnS/NiS_2_ electrodes.

In order to prove that cation-exchange and vulcanization are feasible strategies to improve capacitive properties, a three-electrode system was used to investigate the electrochemical performances of the Zn-BTC, ZnS, Ni-BTC, NiS, Zn/Ni-BTC and ZnS/NiS_2_ electrodes in 1 M KOH aqueous solution at room temperature. Figure 6a exhibits cyclic voltammetry curves of all active electrodes at 20 mV/s. For each sample, the main capacitance can be ascribed to pseudocapacitive behavior from reversible Faradaic reactions since all curves produced a pair of cathode/anode peaks [33]. At the same time, ZnS/NiS_2_ shows the highest current density response compared to other electrodes, indicating that ZnS/NiS_2_ exhibits the highest specific capacitance. The corresponding Faradaic reactions of ZnS/NiS_2_ can be described using the following equations [21,34]:

ZnS + OH^−^←→ZnSOH + e^−^ (4)

ZnSOH + OH^−^←→ZnSO + H_2_O + e^−^ (5)

NiS_2_ + OH^−^←→NiS_2_OH + e^−^ (6)

In an alkaline environment, when a supercapacitor discharges, the nickel sulfide zinc sulfide composite in the positive electrode material combines with hydroxide, respectively, to produce free electrons.

This appearance is clearly evidenced in the presence of abundant electrochemically active sites in the ZnS/NiS_2_ heterostructure. As a consequence, both a rapid electron transfer and a fast reversible Faradaic reaction can be generated. To further study the capacitive performance, Figure 6b presents the GCD curves of all active electrodes at a current density of 1 A/g. The nearly equivalent charge/discharge time and the obviously different voltage platforms indicate satisfactory electrochemical reversibility and excellent Coulombic efficiency. This result is well in agreement with CV shapes. In order to clarify the transport characteristics of the charge carrier, the electrochemical impedance spectroscopy (EIS) was tested. In the Nyquist plot (Figure 6c), all curves are composed of a well-defined semicircle in the high/medium-frequency region and a straight line in the low-frequency region. The semicircles correspond to the charge-transfer in the Faradaic reactions between the active material and the electrolyte. The charge transfer efficiency and ion diffusion efficiency of electrode materials can be expressed via charge transfer resistance (Rct) and diffusion resistance (Rs), respectively. Usually, the smaller the diameter of the semicircle is, the lower the resistance of charge transfer is. The straight line presents the ionic conductivity of the electrolyte. The larger slope of the straight line indicates better conductivity of the electrode materials [35]. Obviously, ZnS/NiS_2_ possesses better conductivity and lower charge transfer resistance as an electrode material than the other samples because it possesses a lower radius and a larger slope. Zn/Ni-BTC in Figure 6d demonstrates the cyclic voltammograms of ZnS/NiS_2_ at various sweep rates. The obvious redox peaks in the curves reveal the pseudocapacitive performance of the ZnS/NiS_2_. Owing to the high internal diffusion resistance, the anode and cathode peaks move to higher and lower voltages, respectively, when increasing the scanning rate. This phenomena is also observed in other systems [36]. Figure 6e shows the galvanostatic charge–discharge (GCD) curves of ZnS/NiS_2_ within the current densities of 1–10 A/g. All the curves feature a pair of charge−discharge plateaus and symmetric shape, indicating the excellent reversibility and high charge–discharge Coulombic efficiency. In addition, no obvious IR was observed, which means there is excellent electronic conductivity and Coulombic efficiency [37]. With the increase in current density, the specific capacitance decreases are due to insufficient redox sites [38]. And GCD curves of Zn-BTC, ZnS, Ni-BTC, NiS and Zn/Ni-BTC are also presented in Figure S4. According to calculation, the specific capacitance of all active electrodes at different charge–discharge current densities is present In Figure 6f. For the ZnS/NiS_2_, the increase in current density (from 1 A/g to 6 A/g) leads to a decrease in specific capacitance (from 1547 F/g to 1214 F/g). In total, 78% of its initial specific capacitance is retained. Compared with the electrode materials reported, it is better than others, such as g-C_3_N_4_/ZnS (497 F/g at 1 A/g) [16], ZnO/Co_3_O_4_ (1135 F/g at 1 A/g) [39], ZnCoS (1134 F/g at 1 A/g) [40] and NiS@NC (1330 F/g at 0.5 A/g) [41]. Moreover, after 4000 charge–discharge cycles (Figure S5), the specific capacitance retention is 70% at 10 A/g. It also can be seen that the specific capacitances of Zn-BTC, ZnS, Ni-BTC, NiS and Zn/Ni-BTC electrodes are 102 F/g, 180 F/g, 828 F/g, 1159 F/g and 1039 F/g at a current density of 6 A/g, respectively, indicating that ZnS/NiS_2_ presents excellent capacitive behavior and rate capability at high current densities. 

**Figure 7:**
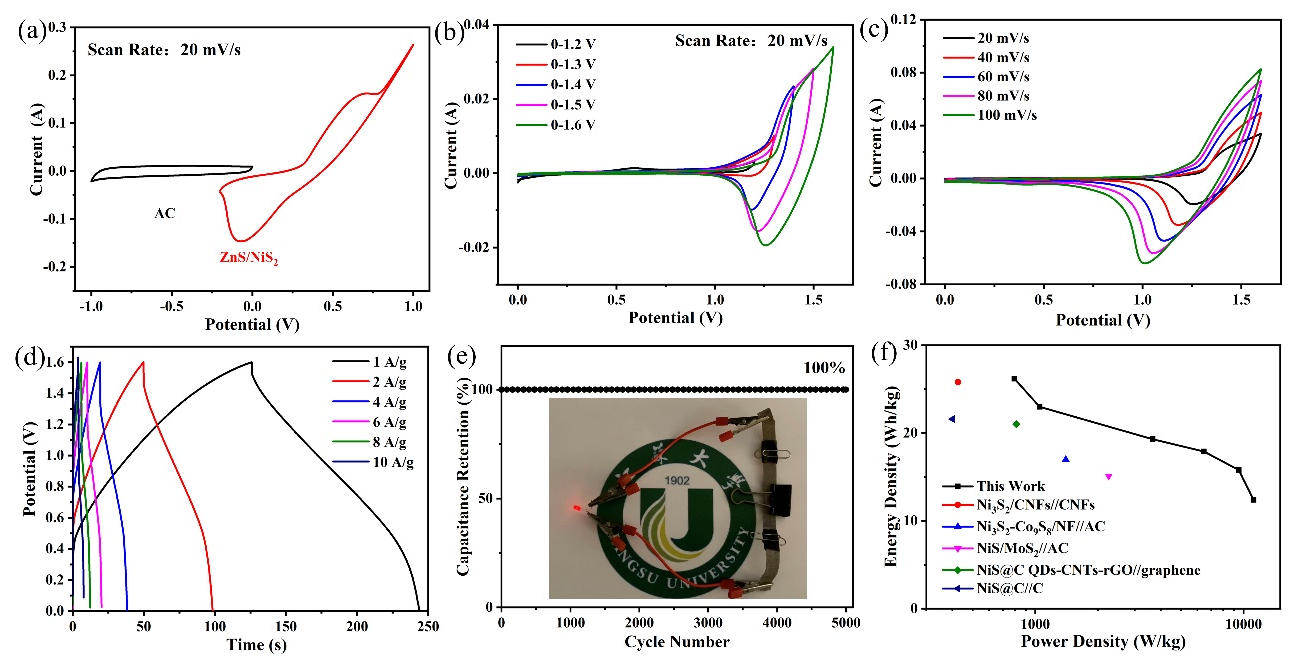
Electrochemical properties of the ZnS/NiS_2_//AC device. (**a**) CV profiles of ZnS/NiS_2_ positive electrode and AC negative electrode at 20 mV/s; (**b**) CV profiles of ZnS/NiS_2_//AC at various voltage windows; (**c**) CV profiles of ZnS/NiS_2_//AC within 20–100 mV/s; (**d**) GCD profiles of ZnS/NiS_2_//AC within 1–10 A/g current density; (**e**) cyclic stability of ZnS/NiS_2_//AC at 10 A/g. Inset shows the optical photographs of the lighted red light-emitting diode (LED) and (**f**) Ragone plot and comparison of energy densities from the literature.

To demonstrate the feasibility of ZnS/NiS_2_ in energy storage devices, we fabricated asymmetric supercapacitor devices by using the ZnS/NiS_2_ as the cathode and the commercial activated carbon (AC) as the anode material (ZnS/NiS_2_//AC). The electrochemical performances of the AC were studied using CV and GCD measurements (Figure S6a,b). The AC showed nearly rectangular shapes and presented a specific capacitance of 121 F/g at a current density of 1 A/g. According to the charge balance, the mass of active materials on the anode and cathode is 1:5. As shown in Figure 7a, corresponding CV curves of AC and ZnS/NiS_2_ showed typical electric double-layer capacitance and obvious pseudocapacitive behavior, respectively [42]. By varying the voltage window and keeping the scan rate at 20 mV/s, a series of CV curves were measured, which demonstrated that the maximum operation potential window could reach up to 1.6 V and could avoid water decomposition effectively (Figure 7b) [43]. The device shows CV curves from 20 to 100 mV (Figure 7c), indicating a good charge mechanism with reversible behavior. Typical GCD curves at different current densities between 1 and 10 A/g in the voltage window of 0−1.6 V are illustrated in Figure 7d, showing a symmetric shape and indicating the good Coulombic efficiency and excellent reversibility of ZnS/NiS_2_//AC. This device achieves a specific capacitance of 73.8 F/g and 35 F/g at current densities of 1 A/g and 10 A/g, respectively (Figure S7). The cyclic stability is carefully studied as one of the important parameters to determine the practicability of the device. The device shows an excellent cycle stability of 100% initial capacity at a current density of 10 A/g after 5000 cycles (Figure 7e). And, we fabricated two asymmetric supercapacitors and lighted a red LED successfully, as shown in the inset of Figure 7e. Finally, a Ragone plot was used for the comparative performance evaluation of the ZnS/NiS_2_//AC ASC device, which exhibited a maximum energy density of 26.3 Wh/kg at a power density of 794 W/kg, with 12.4 Wh/kg remaining at a power density of 1.1 kW/kg, which is higher than what was previously reported (Figure 7f), i.e., Ni_3_S_2_/CNFs//CNFs (25.8Wh/kg at 425 W/kg) [44], Ni_3_S_2_-Co_9_S_8_/NF//AC (17 Wh/kg at 1.4 kW/kg) [45], NiS/MoS_2_//AC (15.1 Wh/kg at 2.25 kW/kg) [46], NiS@CQDs-CNTs-rGO//graphene (21 Wh/kg at 811 W/kg) [47] and NiS@C//C (21.6 Wh/kg at 400 W/kg) [48]. All findings are also compared with the existing literature using data in Table 1. All of these demonstrate the excellent performance of the fabricated asymmetric device.

## 4. Conclusions

In summary, we demonstrated a novel strategy for synthesizing a hierarchical ZnS/NiS_2_ heterostructure through cation exchange and vulcanization using Zn-BTC as the starting precursor. The hierarchical ZnS/NiS_2_ heterostructure can afford abundant electrochemical sites and fast electron transport. Thanks to the heterostructure, the ZnS/NiS_2_ composite electrodes provided ideal pseudocapacitive behavior in KOH aqueous electrolytes. When coupled with AC, the ZnS/NiS_2_//AC hybrid supercapacitor device delivered a wide potential window of 1.6 V and a favorable energy density of 26.3 Wh/kg at a power density of 794 W/kg. Significantly, the capacitance value showed 100% capacitance retention over 5000 charge/discharge cycles. Therefore, the prepared ZnS/NiS_2_ heterostructure is promising candidate for electrodes for high-performance supercapacitors.

## Figures and Tables

**Table 1:** Comparison of energy densities, power densities and specific capacitances from the literature.

Electrode Material	Specific Capacitance (F/g)	Energy Density (Wh/kg)	Power Density (W/kg)	Reference
Ni_3_S_2_/CNFs//CNFs	52	25.8	425	[44]
Ni_3_S_2_-Co_9_S_8_/NF//AC	76.48	17	1400	[45]
NiS/MoS_2_//AC	33	15.1	2250	[46]
NiS@CQDs-CNTs-rGO//graphene	50	21	811	[47]
NiS@C//C	124	21.6	400	[48]
AC//ZnS/NiS2	73.8	26.2	793.8	Our work

## Data Availability

The data presented in this study are available on reasonable request from the corresponding author.

## Supplementary Materials

The following supporting information can be downloaded at: https://www.mdpi.com/article/10.3390/nano14010022/s1, Figure S1: (a) XRD patterns of Zn-BTC, (b) ZnS. The corresponding standard pattern of are presented. Figure S2: (a) XRD patterns of Ni-BTC, (b) NiS. The corresponding standard pattern of are presented. Figure S3: FESEM images of (a) Ni-BTC, (b) NiS and the TEM image of (c) Ni-BTC, (d) NiS. Figure S4: GCD curves of (a) Zn-BTC, (b) ZnS, (c) Ni-BTC, (d) NiS and (e) Zn/Ni-BTC. Figure S5: Cycle performance of the ZnS/NiS_2_. Figure S6: (a) CV curves of AC, (b) GCD curves of AC. Figure S7: The calculated specific capacitance at various current densities.
