# Supplementary material for: Zn-BTC MOF as Self-Template to Hierarchical ZnS/NiS2 Heterostructure with Improved Electrochemical Performance for Hybrid Supercapacitor
Source: Nanomaterials (Basel). 2023 Dec 20;14(1):22. doi: 10.3390/nano14010022 (PMC10780987; doi:10.3390/nano14010022)
Supplement: Supplementary file 1 [file nanomaterials-14-00022-s001.zip › nanomaterials-2747396-supplementary.pdf]

Zn-BTC MOF as Self-Template to Hierarchical ZnS/NiS_2 Heterostructures with Improved Electrochemical Performance for Hybrid Supercapacitor

Xuan Li,[1] Lingran Liu,[1] Chengyu Tu,[1] Quan Zhang,[2,3,*] Xinchun Yang,[3] Daniil I. Kolokolov,[4] Hanna Maltanava,[5] Nikita Belko,[5] Sergey Poznyak,[5] Michael Samtsov,[5] Haixin Guo,[6] Shuping Wu,[1,] Maiyong Zhu[1,*]

1 Research School of Polymeric Materials, School of Materials Science & Engineering, Jiangsu University, Zhenjiang 212013, China; 2211905029@stmail.ujs.edu.cn (X.L.); 2222105022@stmail.ujs.edu.cn (L.L.); 2221905054@stmail.ujs.edu.cn (C.T.); shupingwu@ujs.edu.cn (S.W.)

2 State Key Laboratory for Modification of Chemical Fibers and Polymer Materials, College of Materials Science and Engineering, Donghua University, Shanghai 201620, China

3 Clean Energy Joint International Laboratory, Low-Dimensional Energy Materials Research Center, Shenzhen Institute of Advanced Technology, Chinese Academy of Sciences, Shenzhen 518055, China; xc.yang@siat.ac.cn

4 Boreskov Institute of Catalysis, Siberian Branch of Russian Academy of Sciences, Novosibirsk 630090, Russia; kdi@catalysis.ru

5 Research Institute for Physical Chemical Problems, Belarusian State University, Leningradskaya Str. 14, 220006 Minsk, Belarus; maltanava@bsu.by (H.M.); belkonv@bsu.by (N.B.); poznyak@bsu.by (S.P.); samtsov@bsu.by (M.S.)

6 Agro-Environmental Protection Institute, Ministry of Agriculture and Rural Affairs, No. 31 Fukang Road, Nankai District, Tianjin 300191, China; haixin_g@126.com

* Correspondence: authors: zhangquan@dhu.edu.cn (Q.Z.); maiyongzhu@ujs.edu.cn (M.Z.)

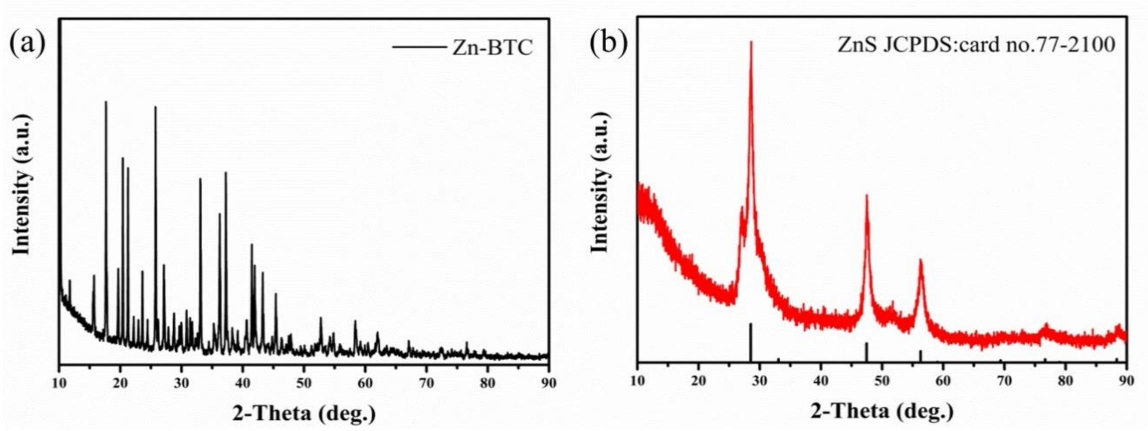

Figure S1. (a) XRD patterns of Zn-BTC, (b) ZnS. The corresponding standard pattern of are presented.

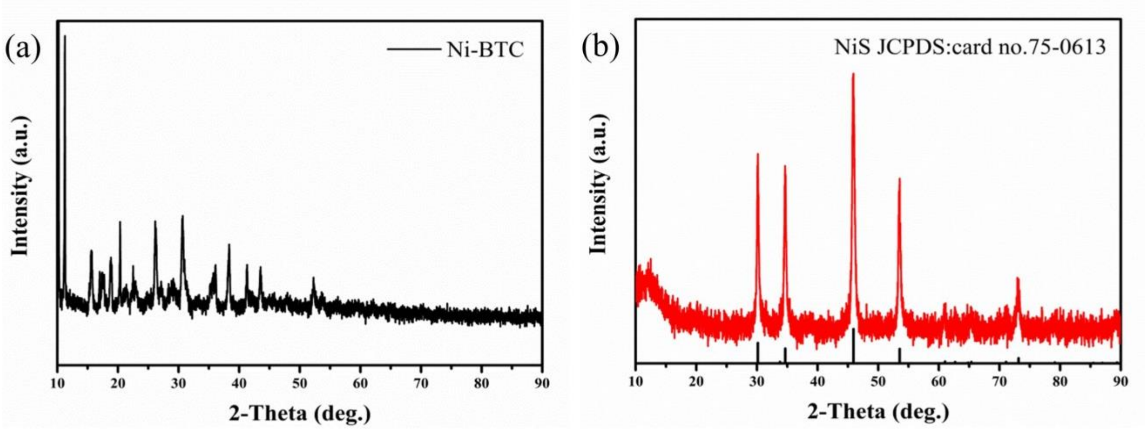

Figure S2. (a) XRD patterns of Ni-BTC, (b) NiS. The corresponding standard pattern of are presented.

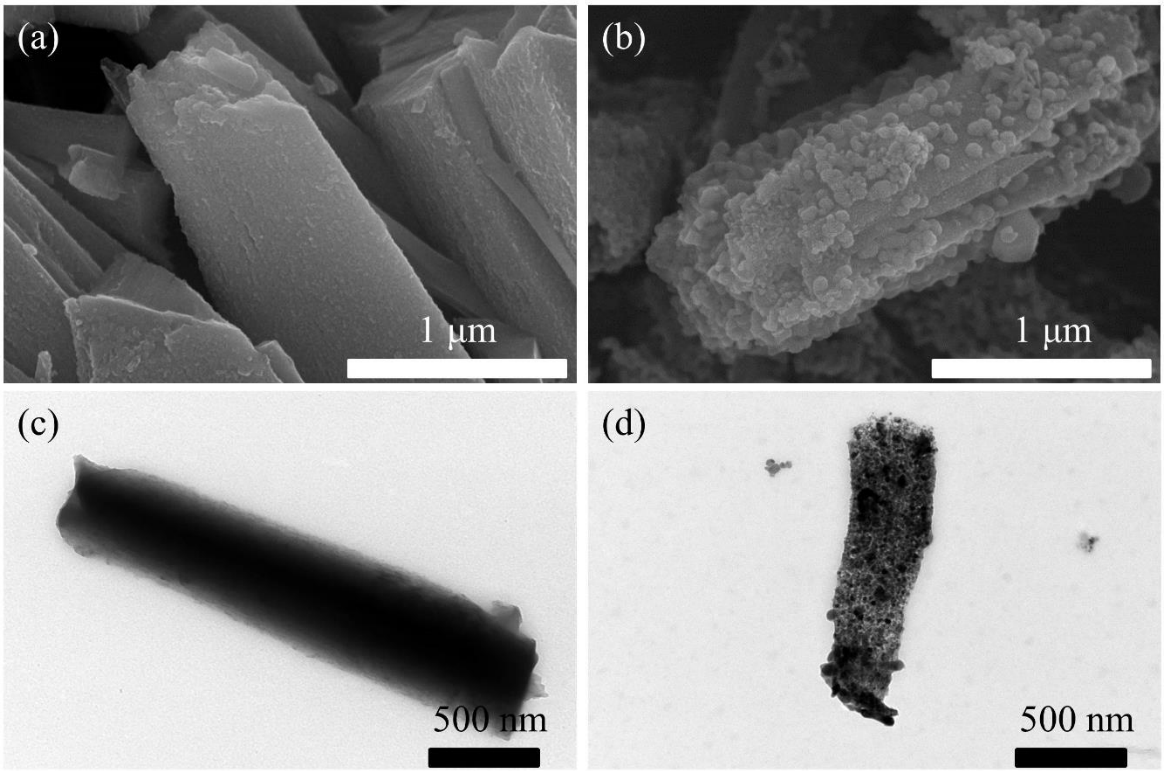

Figure S3. FESEM images of (a) Ni-BTC, (b) NiS and the TEM image of (c) Ni-BTC, (d) NiS.

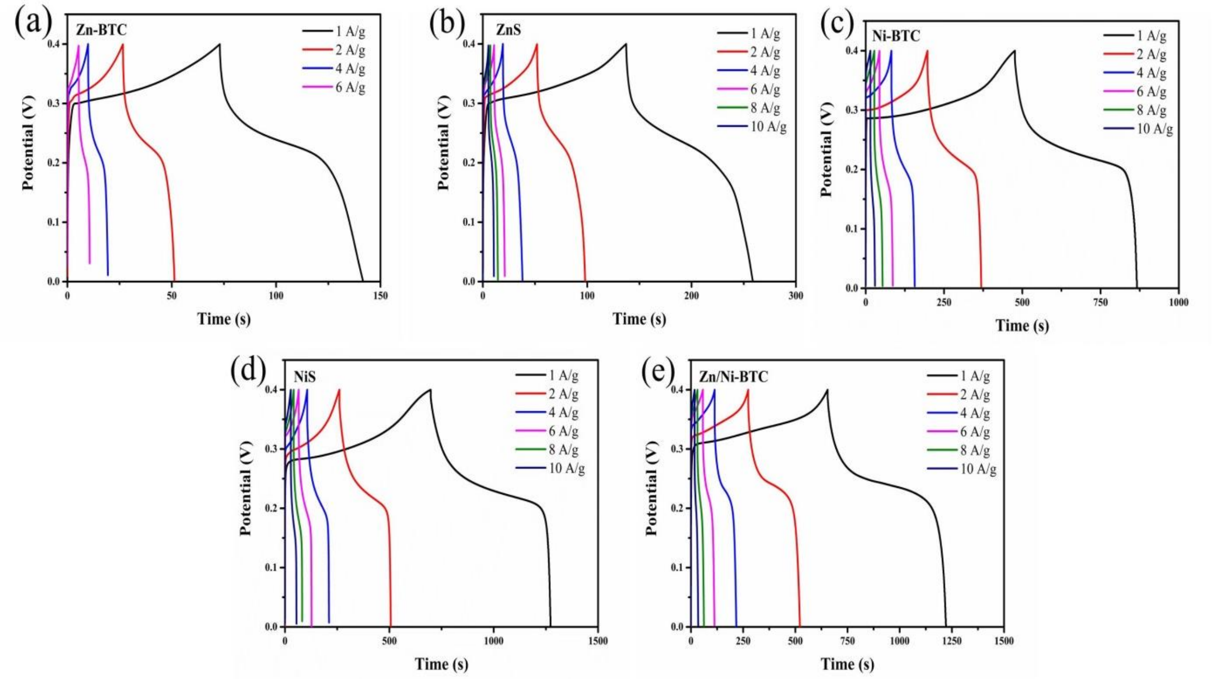

Figure S4. GCD curves of (a) Zn-BTC, (b) ZnS, (c) Ni-BTC, (d) NiS and (e) Zn/Ni-BTC.

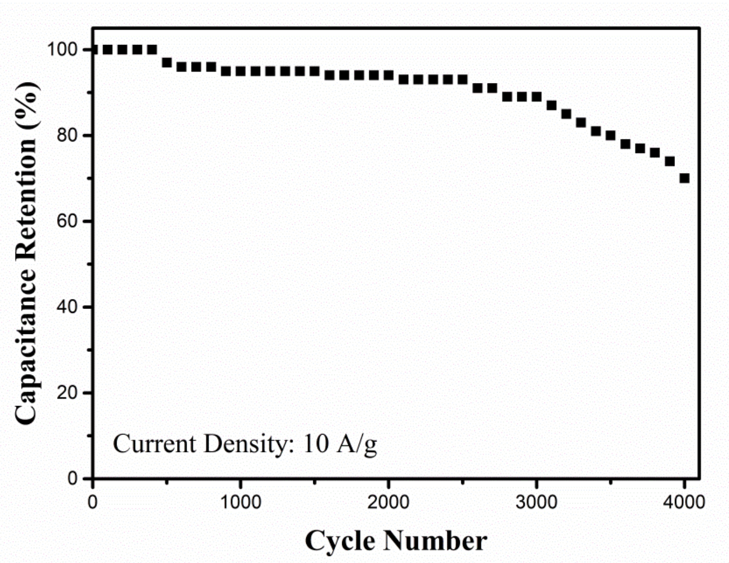

Figure S5. Cycle performance of the ZnS/NiS₂.

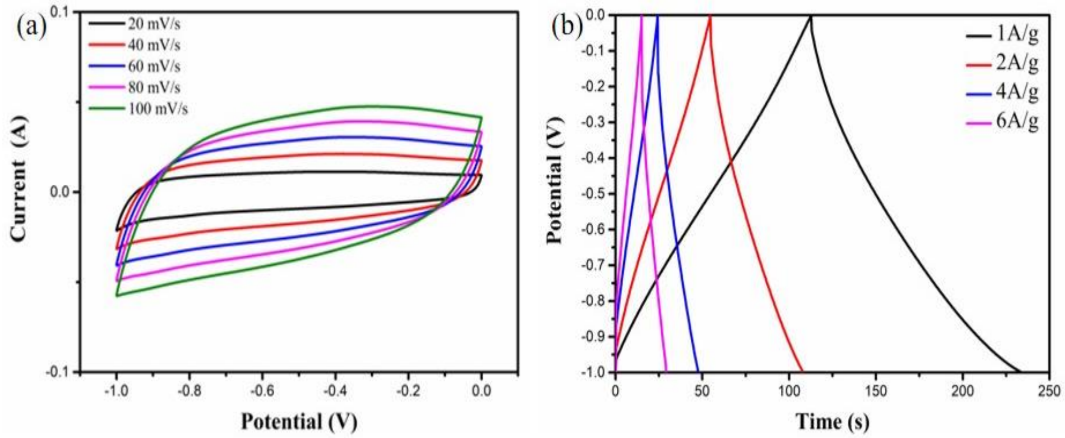

Figure S6. (a) CV curves of AC, (b) GCD curves of AC.

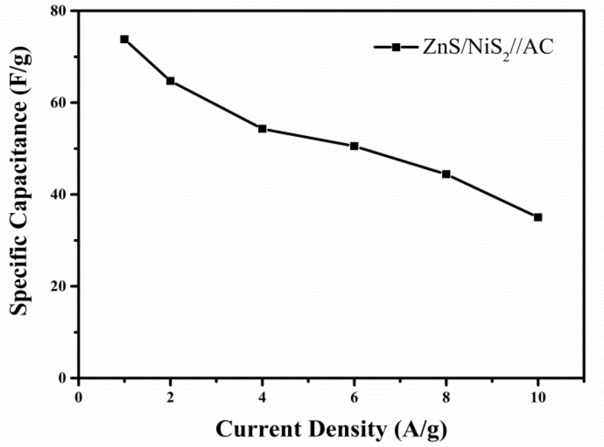

Figure S7. The calculated specific capacitance at various current densities.
